# No significant association of serum klotho concentration with blood pressure and pulse wave velocity in a Chinese population

**DOI:** 10.1038/s41598-021-82258-5

**Published:** 2021-01-27

**Authors:** Wan-Ying Liang, Li-Hong Wang, Jian-Hang Wei, Qing-Lu Li, Qi-Yan Li, Quan Liang, Nai-Qing Hu, Li-Hua Li

**Affiliations:** Department of Gerontology, The First Affiliated Hospital of Dali University, Jiashibo Road 32, Dali, 671000 Yunnan Province China

**Keywords:** Biomarkers, Cardiology, Endocrinology

## Abstract

Klotho, an important anti-aging protein, may be related to elevated blood pressure (BP) and arterial stiffness. We aimed to investigate associations between the serum klotho concentration and peripheral/central BP and arterial stiffness based on the carotid–femoral pulse wave velocity (cfPWV) in a Chinese population. We invited all inhabitants aged ≥ 18 years in two Dali communities for participation. The SphygmoCor system was used to record radial arterial waveforms. Aortic waveforms were derived using a generalized transfer function. The central BP was assessed by calibrating the brachial BP, which was measured using an oscillometric device. The serum klotho concentration was measured using an enzyme-linked immunosorbent assay and logarithmically transformed. Of the 716 participants (mean age: 51.9 ± 12.6 years), 467 (65.2%) were women. The median serum klotho concentration was 381.8 pg/mL. The serum klotho concentration did not significantly differ between patients with and without hypertension (*P* > 0.05) and between those with and without arterial stiffness (cfPWV ≥ 10 m/s) (*P* > 0.05). After adjusting for confounders, the serum klotho concentration was not significantly associated with the peripheral or central BP (*P* > 0.05) and cfPWV (*P* > 0.05). Our data indicated that the serum klotho concentration was not associated with BP or cfPWV in the general Chinese population.

## Introduction

*Klotho* is an anti-aging gene that shortens and extends the lifespan when disrupted and overexpressed, respectively^1^. *Klotho*-deficient mice exhibit signs of accelerated aging, such as arterial stiffness, hypertension, and chronic kidney disease (CKD)^1^. The α-klotho protein encoded by the *klotho* gene is a multifunctional protein that regulates the metabolism of calcium, phosphate, and vitamin D. The following three types of α-klotho protein with potentially different functions have been identified: full-length transmembrane α-klotho, truncated soluble α-klotho, and secreted α-klotho^2^. Previous studies have indicated that the prevalence of hypertension and arterial stiffness increases with age^3,4^, whereas the α-klotho concentrations decrease with age^5^.

A few studies have reported that klotho deficiency is associated with hypertension^6^, salt-sensitive hypertension^7,8^, CKD^9^, arterial stiffness^10,11^, and cardiomyopathy^12^. Therefore, evaluation of circulating klotho concentrations or *klotho* genotypes could help identify patients at higher risk of developing age-related cardiovascular morbidities. However, previous studies on the relationship between circulating klotho and blood pressure and arterial stiffness had small sample sizes^13^, used a case–control design^14^, performed analyses only in select patients^13,15,16^, measured peripheral blood pressure only^8^, or reported conflicting results^15,17^. In a recent study in young and middle-aged swine, elevated klotho secretion was associated with increased aortic stiffness and peripheral vascular resistance with aging^17^. In the present population-based study, we investigated the associations between the serum klotho (i.e., secreted α-klotho) concentrations and the peripheral/central blood pressure and arterial stiffness based on the carotid–femoral pulse wave velocity (cfPWV).

## Results

### Characteristics of the study population

Of the 716 participants, 467 (65.2%), 226 (31.6%), and 76 (10.6%) were women, had hypertension, and had diabetes, respectively. Table 1 presents the participant characteristics according to their sex. Men and women had similar characteristics, except for the rates of current smoking, alcohol consumption, hypertension, and diabetes mellitus, which were higher in men (*P* ≤ 0.01). Men also tended to be older and had a higher body weight, body height, body mass index, waist circumference, hip circumference, fasting levels of blood glucose, low-density lipoprotein cholesterol, triglyceride levels, peripheral systolic and diastolic blood pressures, central systolic and diastolic blood pressures, and cfPWV compared to women. The high-density lipoprotein cholesterol levels and glomerular filtration rate (*P* < 0.0001) were also lower in men than in women. Men and women had similar serum klotho concentrations (442 vs. 365 pg/mL, respectively, *P* = 0.17).

**Table 1:** Characteristics of the study population.

Variable	Men (n = 249)	Women (n = 467)	*P*
Current smoking, n (%)	137 (55.0)	8 (1.7)	< 0.0001
Alcohol intake, n (%)	73 (29.3)	9 (1.9)	< 0.0001
Hypertension, n (%)	101 (40.6)	126 (27.0)	0.0002
Diabetes mellitus, n (%)	40 (16.0)	36 (7.7)	0.0005
Age, years	53.6 ± 13.5	51.0 ± 12.0	0.01
Body height, cm	168.3 ± 5.8	157.1 ± 5.9	< 0.0001
Body weight, kg	69.5 ± 10.9	58.3 ± 8.8	< 0.0001
Body mass index, kg/m^2^	24.5 ± 3.4	23.6 ± 3.3	0.0005
Waist circumference, cm	89.0 ± 9.0	83.1 ± 8.9	< 0.0001
Hip circumference, cm	96.8 ± 7.3	94.6 ± 7.3	0.0002
Total protein, g/L	76.8 ± 7.7	77.4 ± 4.6	0.26
Fasting blood glucose, mmol/L	5.9 ± 1.8	5.5 ± 1.4	0.001
Total cholesterol, mmol/L	4.8 ± 1.0	4.8 ± 0.9	0.86
HDL-C, mmol/L	1.3 ± 0.2	1.4 ± 0.2	< 0.0001
LDL-C, mmol/L	2.7 ± 0.7	2.5 ± 0.7	0.002
Triglycerides, mmol/L	1.7 (1.2–2.4)	1.4 (1.0–1.9)	0.002
GFR, mL/min/1.73 m^2^	87.0 ± 18.0	95.3 ± 18.7	< 0.0001
Serum klotho, pg/mL	442 (199–921)	365 (168–995)	0.17
Peripheral SBP, mmHg	122.4 ± 17.5	117.0 ± 17.7	0.0001
Peripheral DBP, mmHg	79.7 ± 11.8	75.9 ± 10.8	< 0.0001
Peripheral PP, mmHg	42.8 ± 12.0	41.2 ± 11.0	0.07
Central SBP, mmHg	118.9 ± 15.8	114.2 ± 17.2	0.0003
Central DBP, mmHg	81.7 ± 12.1	76.5 ± 11.4	< 0.0001
Central PP, mmHg	36.7 ± 8.5	37.1 ± 9.5	0.55
Pulse rate, beats/min	71.2 ± 9.3	72.4 ± 8.5	0.09
cfPWV, m/s	11.2 ± 2.2	10.0 ± 1.8	< 0.0001

Values are presented as means ± standard deviations, medians (interquartile ranges), or numbers (%).

*cfPWV* carotid–femoral pulse wave velocity, *DBP* diastolic blood pressure, *GFR* glomerular filtration rate, *HDL-C* high-density lipoprotein cholesterol, *LDL-C* low-density lipoprotein cholesterol, *PP* pulse pressure, *SBP* systolic blood pressure.

Table 2 summarizes the study population characteristics stratified by the presence or absence of hypertension. Participants with and without hypertension had similar serum klotho concentrations, body height, lipid profile, and pulse rate (*P* > 0.05). However, compared with normotensive participants, those with hypertension included fewer women and had lower glomerular filtration rates; higher rates of current smoking, alcohol intake, diabetes mellitus; older age; and higher body weight, body mass index, waist and hip circumference, total protein levels, peripheral and central blood pressures, and cfPWV (*P* ≤ 0.03).

**Table 2:** Characteristics of the study population stratified by the hypertension status.

Variable	Hypertensive (n = 227)	Normotensive (n = 489)	*P*
Women, n (%)	126 (55.5)	341 (69.7)	0.0002
Current smoking, n (%)	57 (25.1)	88 (18.0)	0.03
Alcohol intake, n (%)	38 (16.7)	44 (9.0)	0.003
Diabetes mellitus, n (%)	39 (17.2)	37 (7.6)	0.0001
Age, years	58.3 ± 10.4	48.9 ± 12.4	< 0.0001
Body height, cm	161.3 ± 8.2	160.1 ± 7.8	0.48
Body weight, kg	66.3 ± 11.4	60.3 ± 10.2	< 0.0001
Body mass index, kg/m^2^	25.4 ± 3.4	23.2 ± 3.1	< 0.0001
Waist circumference, cm	89.7 ± 8.8	83.0 ± 8.9	< 0.0001
Hip circumference, cm	98.6 ± 7.2	93.9 ± 7.0	< 0.0001
Total protein, g/L	78.1 ± 7.7	76.8 ± 4.8	0.02
Fasting blood glucose, mmol/L	5.9 ± 1.8	5.5 ± 1.4	0.002
Total cholesterol, mmol/L	4.9 ± 1.0	4.8 ± 0.9	0.19
HDL-C, mmol/L	1.4 ± 0.2	1.4 ± 0.2	0.31
LDL-C, mmol/L	2.6 ± 0.7	2.6 ± 0.7	0.40
Triglycerides, mmol/L	1.8 (1.3–2.4)	1.4 (1.0–1.9)	0.002
GFR, mL/min/1.73 m^2^	87.1 ± 20.5	94.8 ± 17.6	0.006
Serum klotho, pg/mL	382 (185–947)	382 (178–952)	0.52
Peripheral SBP, mmHg	135.5 ± 17.2	111.2 ± 12.0	< 0.0001
Peripheral DBP, mmHg	86.4 ± 11.7	72.9 ± 8.2	< 0.0001
Peripheral PP, mmHg	49.1 ± 12.9	38.3 ± 8.7	< 0.0001
Central SBP, mmHg	128.8 ± 18.2	109.8 ± 12.1	< 0.0001
Central DBP, mmHg	87.3 ± 12.7	74.2 ± 8.8	< 0.0001
Central PP, mmHg	41.0 ± 12.1	35.1 ± 6.7	< 0.0001
Pulse rate, beats/min	72.6 ± 9.1	71.7 ± 8.6	0.20
cfPWV, m/s	11.9 ± 2.1	9.7 ± 1.5	< 0.0001

Values are presented as means ± standard deviations, medians (interquartile ranges), or numbers (%).

*cfPWV* carotid–femoral pulse wave velocity, *DBP* diastolic blood pressure, *GFR* glomerular filtration rate, *HDL-C* high-density lipoprotein cholesterol, *LDL-C* low-density lipoprotein cholesterol, *PP* pulse pressure, *SBP* systolic blood pressure.

### Univariate analyses

The median serum klotho concentration was 382 pg/mL. The serum klotho concentration was not significantly associated with the peripheral blood pressure, central blood pressure, pulse rate, or cfPWV (*P* > 0.05).

### Peripheral and central blood pressure in relation to the serum klotho concentration

**Figure 1:**
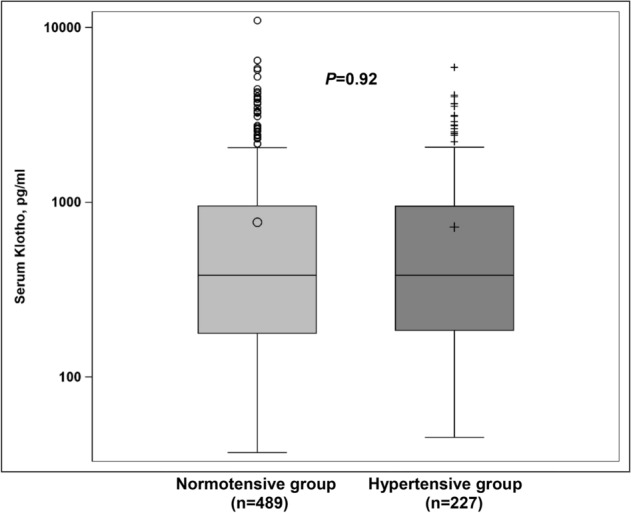
Differences in the serum klotho concentration between patients with and without hypertension.

In the unadjusted analyses, no significant differences were found in the peripheral or central blood pressure levels across the quartiles of the serum klotho concentration (*P* > 0.05). Further adjustments for conventional cardiovascular risk factors including sex, age, body mass index, glomerular filtration rate, hypertension, diabetes mellitus, antihypertensive treatment, current smoking, and alcohol intake did not affect these results (*P* > 0.05) (Table 3). There was no significant difference in the serum klotho concentration between participants with and without hypertension (Fig. 1).

**Table 3:** Peripheral and central blood pressure in relation to the serum klotho concentration.

Characteristics	Klotho groups				*P*
	1st quartile (n = 179) (37–178 pg/mL)	2nd quartile (n = 179) (181–382 pg/mL)	3rd quartile (n = 179) (382–952 pg/mL)	4th quartile (n = 179) (952–10,957 pg/mL)	
**Unadjusted**					
Peripheral SBP, mmHg	118.0 ± 1.3	119.0 ± 1.3	120.6 ± 1.3	118.1 ± 1.3	0.49
Peripheral DBP, mmHg	76.1 ± 0.8	78.3 ± 0.8	78.0 ± 0.8	76.5 ± 0.8	0.18
Peripheral PP, mmHg	41.9 ± 0.8	40.7 ± 0.8	42.6 ± 0.8	41.6 ± 0.8	0.44
Central SBP, mmHg	115.1 ± 1.3	115.9 ± 1.3	115.5 ± 1.3	116.8 ± 1.3	0.82
Central DBP, mmHg	77.2 ± 0.9	79.4 ± 0.9	78.0 ± 0.9	78.77 ± 0.9	0.33
Central PP, mmHg	37.4 ± 0.7	36.0 ± 0.7	37.0 ± 0.7	37.5 ± 0.7	0.36
Adjusted*					
Peripheral SBP, mmHg	118.6 ± 1.0	118.9 ± 1.0	120.2 ± 1.0	118.0 ± 1.0	0.40
Peripheral DBP, mmHg	76.7 ± 0.7	77.8 ± 0.7	77.8 ± 0.7	76.5 ± 0.7	0.37
Peripheral PP, mmHg	41.9 ± 0.7	41.1 ± 0.7	42.4 ± 0.7	41.4 ± 0.7	0.57
Central SBP, mmHg	115.8 ± 1.0	115.8 ± 1.0	115.2 ± 1.0	116.5 ± 1.0	0.84
Central DBP, mmHg	77.8 ± 0.7	79.1 ± 0.7	77.8 ± 0.7	78.7 ± 0.7	0.49
Central PP, mmHg	37.4 ± 0.6	36.2 ± 0.6	36.9 ± 0.6	37.3 ± 0.6	0.52

Values are presented as means ± standard errors. *Values are adjusted for age, sex, body mass index, glomerular filtration rate, hypertension, diabetes status, antihypertensive treatment, current smoking, and alcohol intake.

*DBP* diastolic blood pressure, *PP* pulse pressure, *SBP* systolic blood pressure.

### cfPWV in relation to the serum klotho concentration

**Figure 2:**
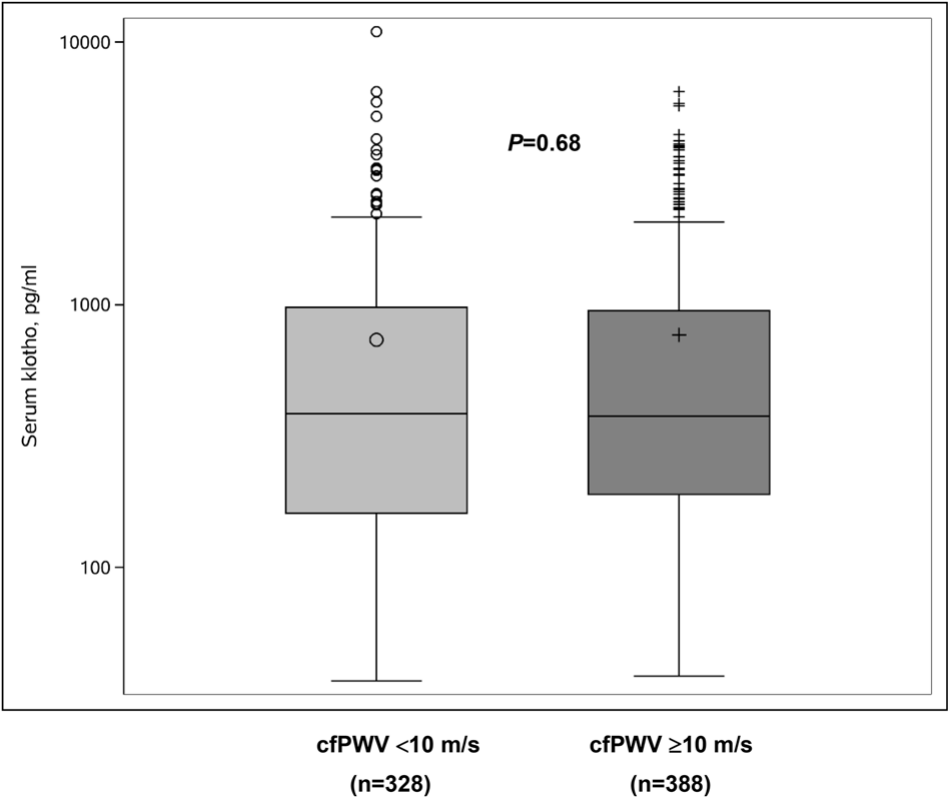
Differences in the serum klotho concentration between participants with cfPWV ≥ 10 m/s and those with cfPWV < 10 m/s. *cfPWV* carotid–femoral pulse wave velocity.

Irrespective of the adjustments for conventional cardiovascular risk factors, there were no significant differences in cfPWV across the quartiles of the serum klotho concentration (*P* > 0.05) (Table 4). There was no significant difference in the serum klotho concentration between the participants with and without arterial stiffness defined by cfPWV (Fig. 2).

**Table 4:** Pulse wave velocity in relation to the serum klotho concentration.

Characteristics	Klotho groups				*P*
	1st quartile (n = 179) (37–178 pg/mL)	2nd quartile (n = 179) (181–382 pg/mL)	3rd quartile (n = 179) (382–952 pg/mL)	4th quartile (n = 179) (952–10,957 pg/mL)	
**Unadjusted**					
cfPWV, m/s	10.4 ± 0.2	10.4 ± 0.2	10.4 ± 0.2	10.5 ± 0.2	0.93
Adjusted*					
cfPWV, m/s	10.4 ± 0.1	10.4 ± 0.1	10.2 ± 0.1	10.5 ± 0.1	0.38

Values are presented as means ± standard errors. *Values are adjusted for age, sex, body height, pulse rate, glomerular filtration rate, hypertension, diabetes status, antihypertensive treatment, current smoking, and alcohol intake.

*cfPWV* carotid–femoral pulse wave velocity.

## Discussion

To the best of our knowledge, this is the first study that systematically investigated the relationship between the serum klotho concentration and blood pressure/arterial stiffness using a population-based design. Our results indicated that the serum klotho concentration is not associated with peripheral or central blood pressure or indices of arterial stiffness.

Few previous studies have indicated that a lower circulating klotho concentration was associated with higher blood pressure or was a risk factor of hypertension. Especially, in an analysis of 79 and 30 older adults with and without hypertension, respectively, Su et al*.* reported that klotho protein absorbance (0.303 ± 0.096) was lower in the hypertensive than in the non-hypertensive group (0.489 ± 0.216)^13^. In a 1:1 matched case–control study (mean age: 51.7 years) of 197 Chinese patients with hypertension and 197 without hypertension, Zhou et al.^14^ reported that the median serum klotho concentrations were 269.67 and 313.95 ng/L (*P* = 0.004) in the hypertensive and normotensive groups, respectively. After adjustments for covariates including age, lifestyle, family history of hypertension, body mass index, fasting blood glucose, serum uric acid, lipid levels, and microalbumin, the risks (odds ratio, OR [95% confidence interval, CI]) of hypertension in the lowest three quartile groups were 2.01 (1.08–3.71), 2.29 (1.20–4.39), and 1.55 (0.82–2.92) compared with those in the highest quartile group, respectively^14^.

In contrast to the results of some previous studies, our findings did not support an association between the serum klotho concentration and blood pressure in the general population^13,14^. However, our results were consistent with those of a larger population-based study^18^. In the InCHIANTI study of 1,203 Italian adults, the median (25th–75th percentile) plasma klotho concentration was 676 (530–819) pg/mL. Plasma klotho was correlated with age but not with systolic blood pressure^18^. Despite convincing experimental evidence regarding the role of klotho deficiency in the pathogenesis of hypertension and salt hypertension^6,19–21^, the roles of klotho in human blood pressure regulation and pathogenesis of hypertension remain largely unknown. The discrepancies between the present and previous results may be attributable to differences in the study population age^13^ or small sample sizes of previous studies (≤ 394 participants)^13,14^. Furthermore, it is also possible that klotho is important in regulating blood pressure only in those processes or pathologies that are associated with a very drastic reduction in its serum concentrations. Further large-scale prospective studies are required to verify the relationships between the circulating klotho levels and blood pressure changes and the risk of hypertension in different age groups and disease conditions.

Few previous studies have investigated the relationship between circulating klotho and arterial stiffness based on baPWV or cfPWV measurements. Our finding that the serum klotho concentration was not associated with arterial stiffness in the general Chinese population is consistent with the results of most, but not all, previous studies^15,16,22,23^. A previous study in 2101 participants from the KoreaN Cohort Study for Outcome in Patients With Chronic Kidney Disease (KNOW-CKD) cohort reported no significant association between the serum klotho concentration and baPWV after adjustments (β = 0.003; 95% CI: − 0.04–0.05; *P* = 0.876)^16^. In 109 patients with diabetic nephropathy, pulse wave velocity (PWV) was reported to increase in those with CKD but was not related to serum klotho concentration^22^. However, our results contradicted those reported in some small-scale studies^15,23^ and in studies that included patients undergoing hemodialysis^23^ or those with CKD^15^. In a case–control study of 130 age- and sex-matched patients undergoing hemodialysis, α-klotho concentrations were inversely associated with the aortic–brachial PWV ratio (β =  − 0.070; 95% CI: − 0.133 to − 0.006)^23^. In another study that included 114 patients with CKD, there were significant decreases in the serum klotho concentration in those with baPWV ≥ 1400 cm/s^15^.

A growing body of evidence has suggested that some single nucleotide polymorphisms in the *klotho* gene were correlated with the circulating klotho levels and the susceptibility to hypertension^24^, salt hypertension^8^, and coronary artery disease^25^ as well as the onset of stroke^26^. Though we did not find a significant relationship between the serum klotho concentrations, blood pressure, and PWV, we cannot exclude the possibility that genetic polymorphisms of the *klotho* gene may be associated with blood pressure and arterial stiffness.

Although our study did not support a relationship between the serum klotho and blood pressure and arterial stiffness in middle-aged Chinese individuals, previous experimental studies have indicated that klotho deficiency affected the regulation of blood pressure and pathogenesis of arterial stiffness via promoting oxidative stress, inflammation, apoptosis, and fibroblast growth factor receptor (FGFR)/FGF23 resistance^11,27^. Klotho can function as a humoral factor and regulate nitric oxide production in the endothelium, thus, preserving endothelial permeability, calcium homeostasis in the kidneys, and inhibiting insulin-like growth factor-1 signaling^28^. It can also serve as a mediator for the actions of FGF23, namely urinary phosphate excretion, inhibition of calcitriol secretion, and inhibition of parathyroid hormone synthesis and secretion^29^. Klotho deficiency increases NADPH oxidase activity and superoxide production, collagen expression, and elastin fragmentation in the aortic media^30^. Furthermore, klotho deficiency causes salt-sensitive hypertension and renal damage in mice by CC chemokine receptor 2-mediated inflammation^7^.

Our findings clarified and complicated the understanding of the relationship between klotho and blood pressure/arterial stiffness. We measured the serum klotho concentrations, peripheral and central blood pressures, and cfPWV in a much larger population-based sample compared with previous studies, thus, increasing the possibility of identifying any significant associations. However, klotho concentration, blood pressure, and arterial stiffness were highly dependent on age and other cardiovascular risk factors, such as diabetes mellitus, cigarette smoking, CKD, and metabolic syndrome^31–34^. When compared with aging, cigarette smoking, diabetes mellitus, CKD, and other typical cardiovascular risk factors, klotho deficiency may be a relatively weaker risk factor for cardiovascular disease in humans. Further prospective follow-up studies with larger sample sizes are required to elucidate the relationships between circulating klotho, blood pressure, and arterial stiffness.

Our findings should be interpreted within the context of the strengths and limitations of this study. Our study included 716 individuals who underwent comprehensive assessments of central blood pressure and cardiovascular function. However, it presented the following limitations. First, the cross-sectional design did not allow us to make any inferences regarding causality. The current design of our study did not allow us to test the association of klotho deficiency with microalbuminuria and the predictive value for renal function decline^35,36^. Second, the serum klotho concentration was estimated only once, which can be influenced by several factors and may fluctuate with time. Third, the serum was stored at – 30 ℃, and we could not exclude the possibility that the stability of serum klotho decreases with longer storing time^37^. Fourth, although we had very detailed demographic information of each individual, the data regarding antihypertensive medications, which might alter the serum klotho levels^38^, were not available. Nonetheless, the self-reported antihypertensive treatment rate was 19.6%, suggesting that the antihypertensive treatment rate was very low in this study. Further adjustment for antihypertensive treatment did not significantly change our results. Similar results were found in the sensitivity analysis among those not receiving antihypertensive treatment. Fifth, we did not measure the vitamin D and FGF23 levels. The molecular interactions of FGF23, klotho, and vitamin D coordinate to regulate the delicate phosphate levels of human body. Vitamin D can induce FGF23 and klotho synthesis to influence renal phosphate balance^39^. Thus, we could not exclude the possibility that vitamin D and FGF23 could affect the blood pressure regulation and pathogenesis of arterial stiffness. Sixth, the reproducibility of cfPWV measurement was not tested. Nonetheless, the cfPWV measurement was well standardized, and the same technician performed all the cfPWV measurements in this study. Thus, this limitation did not affect our results. Finally, our participants had a relatively lower profile of cardiovascular risk than those included in previous clinical studies. Future large-scale prospective studies should include participants at higher risk to explore the relationships between the serum klotho concentration, blood pressure, hypertension, and arterial stiffness.

In conclusion, our findings demonstrated that the serum klotho concentration was not associated with peripheral/central blood pressure or arterial stiffness in the general Chinese population. Nonetheless, future prospective studies should investigate the importance of circulating klotho measurements in cardiovascular risk stratification.

## Methods

### Study participants

This cross-sectional analysis was based on data collected as part of an ongoing population study of multiple cardiovascular risk factors in Dali, Yunnan Province, China. The study participants were recruited from two communities in Dali. Between October and December 2018, we cooperated with the medical staff of the local community health service center to invite all inhabitants aged ≥ 18 years to participate in the study through notice and telephone calls. Of those invited, 764 (70%) participated. We excluded 48 individuals from our analysis because they did not have blood samples (n = 4) or arterial (n = 39) and data (n = 5). Therefore, a total of 716 participants were included in the present analysis.

The Ethics Committee of Dali University approved the study protocol, and all participants provided written informed consent. All procedures were performed in accordance with the Declaration of Helsinki.

### Field work

After each participant had rested for at least 5 min in the sitting position, two experienced physicians measured the blood pressure five consecutive times using a mercury-based sphygmomanometer. These five readings were averaged for the final analysis. The same physicians also administered a standardized questionnaire to collect information related to the medical history, smoking habits, alcohol intake, and medications. Hypertension was defined as a peripheral systolic or diastolic blood pressure of at least 140 and 90 mmHg, respectively, while seated^40^. Patients who were prescribed antihypertensive drugs were also considered to have hypertension^40^. A trained physician performed the anthropometric measurements. The body mass index was calculated as the body weight in kilograms divided by the body height in meters squared.

Venous blood samples were drawn after overnight fasting for the measurement of plasma glucose and serum total cholesterol and other biochemical analyses. The glomerular filtration rate (GFR) was estimated using the Modification of Diet in Renal Disease Study equation^41^. Diabetes mellitus was defined as a fasting plasma glucose level of at least 7.0 mmol/L, hemoglobin A1c level of at least 6.5%, or the use of antidiabetic agents^42^.

### Central blood pressure and cfPWV measurement

To ensure a steady state, a trained physician performed all arterial measurements via applanation tonometry after participants had rested for 15 min in the supine position. The participants were instructed to refrain from smoking, vigorous exercise, and drinking alcohol or caffeinated beverages for at least 2 h before the examination. We used a high-fidelity SPC-301 micromanometer (Millar Instruments, Houston, TX, USA) interfaced with a laptop computer running SphygmoCor software v7.1 (AtCor Medical, West Tyde, New South Wales, Australia) to record the arterial waveforms. The recordings were discarded when the variability in consecutive waveforms exceeded 5% or when the amplitude of the pulse wave signal was < 80 mV. We calibrated the pulse wave based on the average of two consecutive brachial blood pressure readings obtained with a participant in the supine position immediately prior to SphygmoCor recordings using a validated Omron HEM-7051 oscillometric blood pressure monitor (Omron, Kyoto, Japan). The SphygmoCor software calculates the aortic pulse wave from the radial signal using a validated generalized transfer function. The central systolic and diastolic blood pressures were derived from the aortic pulse wave^43,44^.

For the cfPWV measurement, the physician recorded the right carotid and femoral waveforms (12 s each) in succession. Based on simultaneously recorded electrocardiogram data (lead 2), the time delay between the feet of the two pressure waveforms was taken as the transit time between the carotid and femoral pressure waves. The distance traveled by the pressure wave was determined based on the difference between the distances from the sternal notch to the femoral location and from the sternal notch to the carotid location. PWV was calculated as the distance traveled divided by the transit time^43,44^. Arterial stiffness was defined as a cfPWV ≥ 10 m/s^45^.

### Serum klotho measurements

Serum samples were stored at − 30 °C prior to measurements. The serum klotho concentration was measured using the enzyme-linked immunosorbent assay method in accordance with manufacturer instructions (DY5334-05, R&D Systems, Inc., Minneapolis, MN, USA). The detection range was 78.10–5000 pg/mL. The limit of the kit’s sensitivity was 50 pg/mL.

### Statistical analysis

We used SAS v9.4 (SAS Institute, Cary, NC, USA) for database management and statistical analyses. Normality of data was assessed using the Shapiro–Wilk test. The serum klotho concentration exhibited a non-normal distribution and was, therefore, logarithmically transformed for statistical analysis. The means and proportions were compared using Student’s *t*-tests and Fisher’s exact tests, respectively. We performed unadjusted and multivariate-adjusted variance analyses to investigate the associations of serum klotho concentration with the central and peripheral blood pressures and arterial stiffness. *P* values < 0.05 were considered statistically significant.

## Data Availability

No datasets were generated or analyzed during the current study.
